# Characterization of Novel Salt-Tolerant Esterase Isolated from the Marine Bacterium *Alteromonas* sp. 39-G1

**DOI:** 10.4014/jmb.1907.07057

**Published:** 2019-12-02

**Authors:** Seok-Jae Won, Han Byeol Jeong, Hyung-Kwoun Kim

**Affiliations:** Department of Biotechnology, The Catholic University of Korea, Bucheon 14662, Republic of Korea

**Keywords:** *Alteromonas*, family IV esterase, salt tolerance

## Abstract

An esterase gene, *estA1*, was cloned from *Alteromonas* sp. 39-G1 isolated from the Beaufort Sea. The gene is composed of 1,140 nucleotides and codes for a 41,190 Da protein containing 379 amino acids. As a result of a BLAST search, the protein sequence of esterase EstA1 was found to be identical to *Alteromonas* sp. esterase (GenBank: PHS53692). As far as we know, no research on this enzyme has yet been conducted. Phylogenetic analysis showed that esterase EstA1 was a member of the bacterial lipolytic enzyme family IV (hormone sensitive lipases). Two deletion mutants (Δ20 and Δ54) of the esterase EstA1 were produced in *Escherichia coli* BL21 (DE3) cells with part of the N-terminal of the protein removed and His-tag attached to the C-terminal. These enzymes exhibited the highest activity toward *p*-nitrophenyl (*p*NP) acetate (C_2_) and had little or no activity towards *p*NP-esters with acyl chains longer than C6. Their optimum temperature and pH of the catalytic activity were 45°C and pH 8.0, respectively. As the NaCl concentration increased, their enzyme activities continued to increase and the highest enzyme activities were measured in 5 M NaCl. These enzymes were found to be stable for up to 8 h in the concentration of 3-5 M NaCl. Moreover, they have been found to be stable for various metal ions, detergents and organic solvents. These salt-tolerant and chemical-resistant properties suggest that the enzyme esterase EstA1 is both academically and industrially useful.

## Introduction

Esterase (E.C. 3.1.1.1) and lipase (E.C. 3.1.1.3) are industrial enzymes that catalyze hydrolysis and the transesterification reactions of fatty acid esters [1]. These enzymes are serine hydrolase with serine amino acids at the active site. Many serine hydrolases have been studied for their 3D protein structures, and most enzymes are known to share a common structure, the α/β hydrolase fold [2]. The active site has a catalytic triad (Ser-His-Asp/Glu), and the active serine is in the well-conserved pentapeptide sequence (Gly-x-Ser-x-Gly) [3] and is considered to perform nucleophilic attacks during enzyme reaction as part of the nucleophilic elbow [4]. Esterase prefers hydrolyzing ester bonds of fatty acids with short carbon chains, and lipase prefers hydrolyzing ester bonds of fatty acids with long carbon chains [5].

Esterases and lipases are classified into eight families according to protein sequence, conserved amino acid sequence motif and biochemical property [6]. Among them, some bacterial esterases and lipases are classified as family IV, along with hormone-sensitive lipases (HSL) [7]. Family IV enzymes generally consist of two domains: the cap domain at the N-terminus and the catalytic domain at the C-terminus [8, 9]. Cap domains generally have two α-helices and one loop structure, covering the active site of the catalyst domain. Various family IV esterases and lipases are found in various environments such as hot springs [10], deep seas [11], the Arctic sediments [12], marine sediments [13], and soils [14].

Microorganisms living in extreme environments are a source of industrially useful enzymes [15, 16]. Extreme microorganisms produce extreme enzymes, which have high activity and stability in extreme environments. Interestingly, some extreme enzymes exhibit high activity under more than two extreme conditions [17-19]. The enzymes derived from marine microorganisms have attracted the attention of many researchers because they maintain high enzyme activity even under severe conditions including high temperature, low temperature, and high pH or low pH [20, 21]. In addition, these enzymes have high activity under organic solvents, high salinity and low water activity environments [22], suggesting the potential to be used as industrial biocatalysts [23].

Esterases and lipases have been applied widely in the detergent, biodiesel, food, and pulp and paper industries [24-27]. Lipolytic enzymes derived from *Burkholderia*, for example, are used in biodiesel production [28]. These enzymes can be used for transesterification of waste oils with short chain alcohol in high levels of methanol solvents. Other lipolytic enzymes can be used in biological purification of contaminated environments, especially in low temperature environments, such as oil spills [29].

In this study, an esterase gene (*estA1*) was cloned from *Alteromonas* sp. 39-G1 isolated from the Beaufort Sea. We produced two deletion mutants (Δ20 and Δ54) of the esterase EstA1 and characterized their enzymatic properties in this research.

## Materials and Methods

### Materials

Acetonitrile, 2-propanol, ampicillin, tributyrin, *p*-nitrophenyl (*p*-NP) acetate (C_2_), *p*-NP butyrate (C_4_), *p*-NP caprylate (C_8_), lithium chloride, nickel chloride, sodium dodecyl sulfate, dithiothreitol, 2-mercaptoethanol, Triton X-100 and phenylmethylsulfonyl fluoride were purchased from Sigma Aldrich Co. (USA). Dimethyl sulfoxide, cobalt chloride hexahydrate, magnesium sulfate, potassium chloride, calcium chloride, sodium chloride, zinc chloride, chloroform, cyclohexane, n-hexane and acetone were purchased from Junsei Co. (Japan). *p*-NP caproate (C_6_) was purchased from Tokyo Chemical Industry Co. (Japan). Methanol and ethanol were purchased from Merck Chemical Co. (Germany), and β-D-thiogalactopyranoside was purchased from Duchefa Biochemie B.V. Co. (The Netherlands). Copper sulfate was purchased from Kanto Chemical Co. (Japan). X-gal was purchased from Promega (USA). Ni-NTA agarose was purchased from Qiagen (Germany). We were provided with a lipolytic bacterial strain (No. 39-G1) isolated from the Arctic Beaufort Sea (70-56-1581 N, 136-25-1178 W) by Korea Polar Research Institute.

### Shotgun Cloning of *estA1* Esterase Gene

The chromosomal DNA was extracted from *Alteromonas* sp. 39-G1 strain using a genomic DNA extraction kit (iNtRON Biotechnology, Korea). The isolated chromosomal DNA was digested with *Eco*RI, ligated with pUC19 vector and transformed into *E. coli* DH5α. Among about 10,000 colonies, 13 colonies had lipolytic activity on tributyrin (TBN)-LB agar plate. These colonies were cultured in LB broth and recombinant plasmids were extracted with a LaboPass DNA Mini Kit (Cosmogenetech, Korea). Insert DNAs were sequenced and one functional esterase gene was found and designated as *estA1* gene.

### EstA1 Protein Sequence Analysis and Structure Homology Modeling

The EstA1 protein sequence was predicted from the *estA1* nucleotide sequence using the DNASTAR program. The position and length of the signal sequence of the EstA1 protein was predicted by the SignalP 4.1 Server (http://www.cbs.dtu.dk/services/SignalP-4.1/). The search and similarity calculation of some proteins similar to the EstA1 protein were performed in the BLASTp programs (http://www.ncbi.nlm.nih.gov/BLAST/). Multiple sequence alignment with similar esterase sequences was performed using ClustalW method and aligned sequences were rendered at ESPript 3.0 web (http://espript.ibcp.fr/ESPript/ESPript/). Phylogenetic tree was constructed using the neighbor-joining method with 1000 bootstrap replications implemented by the phylogeny package MEGA 5.2.

The 3D structure of the EstA1 protein was constructed by SWISS-MODEL (https://swissmodel.expasy.org/) using the crystal structure (PDB: 4Q3O) of MGS-MT1, an alpha/beta hydrolase enzyme isolated from a Lake Matapan deep-sea metagenome library as templates and presented using PyMOL program.

### Expression and Purification of the Recombinant Esterase EstA1

Three PCR products (WT, Δ20, and Δ54) amplified by primers (Table S1) were inserted into the pGEM-T vector (Promega, USA). These recombinant plasmids were digested with *Nde*I and *Xho*I and the insert DNA fragments were ligated into pET22 vector. The resulting recombinant plasmids were transformed into *E. coli* BL21 (DE3) cells. Transformed cells were inoculated in 200 ml LB medium containing ampicillin (100 μg/ml) and cultured at 37°C to 230 rpm. The *E. coli* BL21 (DE3) cells were induced to produce the recombinant proteins with 1 mM IPTG at 16°C for 16 h after the OD_600_ reached 0.6. The cells were harvested by centrifugation (4,000 ×g for 10 min) and resuspended in lysis buffer (50 mM NaH_2_PO_4_, 300 mM NaCl, and 10 mM imidazole, pH 8.0), then disrupted by ultrasonicator and the cell-free extract was obtained by centrifugation (12,000 ×g, for 10 min).

The cell-free extract was applied to the Ni-NTA agarose affinity chromatography column that has been previously equilibrated with the lysis buffer. The unbound proteins were removed by washing the column with washing buffer (50 mM NaH_2_PO_4_, 300 mM NaCl, and 20 mM imidazole, pH 8.0) and the esterase EstA1 was eluted with elution buffer (50 mM NaH_2_PO_4_, 300 mM NaCl, and 500 mM imidazole, pH 8.0). The purified esterase EstA1 was loaded onto the FPLC system equipped with a Superpose™ 12 10/300 GL gel permeation column and eluted using elution buffer (50 mM NaH_2_PO_4_ and 300mM NaCl, pH 8.0). Protein concentration was measured by Bradford assay and the purity was analyzed by SDS-PAGE.

### Enzyme Assay

Esterase activity assay was performed with *p*NP-esters as substrates, and the production of *p*-nitrophenol was measured using 405 nm absorbance. One unit of esterase activity was defined as 1 μmol of *p*-nitrophenol liberated per min under standard assay conditions (pH 8.0, 40°C, 3 min). The assay was carried out in 1 ml reaction mixture containing 950 μl of 50 mM Tris-HCl (pH 8.0), 40 μl of ethanol, and 10 μl of 10 mM *p*NP-esters dissolved in acetonitrile solvent.

### Characterization of Esterase EstA1

The substrate specificity of purified esterase EstA1 was determined in standard conditions (pH 8.0, 40°C, 3 min) in 50 mM Tris-HCl buffer containing 10 mM of *p*NP-esters (C_2_-C_8_). The optimal pH for enzyme activity was determined at 40°C in buffers with pH range from 6.0 to 9.5 and *p*NP-butyrate (C_4_) was used as substrate in pH effect assay. The pH stability of the enzyme was tested by measuring the residual activity after 30 min-incubation in various pH buffers; pH 5.0-6.0, 50 mM sodium acetate; pH 6.0-8.0, 50 mM potassium phosphate; pH 8.0-9.0, 50 mM Tris-HCl; pH 9.0-10.5, 50 mM carbonate-bicarbonate. The optimal temperature for enzyme activity was determined over the range of 0-70°C in Tris-HCl buffer (pH 8.0) and *p*NP-butyrate (C_4_) was used as a substrate. The temperature stability of the enzyme was tested by treating enzyme at various temperatures for the indicated time and measuring residual activity.

To investigate the effect of various organic solvents (DMSO, methanol, ethanol, acetonitrile, acetone, isopropanol, cyclohexane, and n-hexane) on the mutant enzymes, the enzymes were incubated in various organic solvents (15, 30, 50%, v/v) for 30 min at 30°C and the residual enzyme activities were measured in standard conditions (pH 8.0, 40°C, 3 min). *p*NP-acetate (C_2_) was used as a substrate. The enzyme activity of the esterase EstA1 solution without solvent was set to 100%.

Enzyme activity was also determined in 1 mM (or 5 mM) metal ions (Li^+^, Na^+^, K^+^, Mg^2+^, Ca^2+^, Co^2+^, Ni^2+^, Zn^2+^, and Cu^2+^), 1 mM (or 5 mM) inhibitors (dithiothreitol, β-mercaptoethanol, glutathione disulfide, EDTA, phenylmethylsulfonyl fluoride), and 0.1% (or 0.5%) detergents (sodium dodecyl sulfate, Tween-20, Tween-40, Tween-80, and Triton X-100). *p*NP-acetate (C_2_) was used as a substrate.

The effect of NaCl on enzyme activity was determined at 40°C in Tris-HCl buffer (pH 8.0). Increasing concentrations (0 to 5 M) of NaCl were used and *p*NP-acetate (C_2_) was used as a substrate. To investigate its stability toward the high concentration of NaCl, enzyme was incubated in Tris-HCl buffer (pH 8.0) containing high concentration (3, 4, and 5 M) of NaCl for 8 h and the residual enzyme activities were measured.

In all experiments, we performed enzyme activity measurements three times and calculated the mean value and presented the standard deviation in the figures.

## Results and Discussion

### Strain Identification

Based on the 16S rRNA sequence analysis (Fig. S1), the strain 39-G1 was classified into the genus *Alteromonas* and designated as *Alteromonas* sp. 39-G1. The phylogenetic tree created by the Clustal W method of the DNASTAR program revealed the same classification (Fig. S2).

### Esterase EstA1 Gene Cloning

The genomic DNA was extracted from *Alteromonas* sp. 39-G1 and DNA fragments were inserted into the pUC19 plasmid and the recombinant plasmid library was transformed into *E. coli* DH5α. Clones showing clear halo in tributyrin/LB agar plate were found, and the recombinant plasmids obtained from the clones were analyzed. The sequence analysis revealed that one open reading frame (ORF), which is presumed to be an esterase/lipase gene, was found.

### Sequence Analysis and Structural Modeling of Esterase EstA1

The ORF consists of 1,140 nucleotides and codes 379 amino acids (Fig. S3). A BLAST search revealed that this protein belongs to the esterase family IV. We decided to call this enzyme ‘esterase EstA1.’ The phylogenetic tree was constructed using this esterase EstA1 and other esterases belonging to family IV (Fig. S4), and this esterase EstA1 was found to be identical with *Alteromonas* sp. esterase (GenBank PHS53692.1). Although this protein sequence has already been reported, we have decided to study it because there has been no report on this protein as far as we know.

**Fig. 1:**
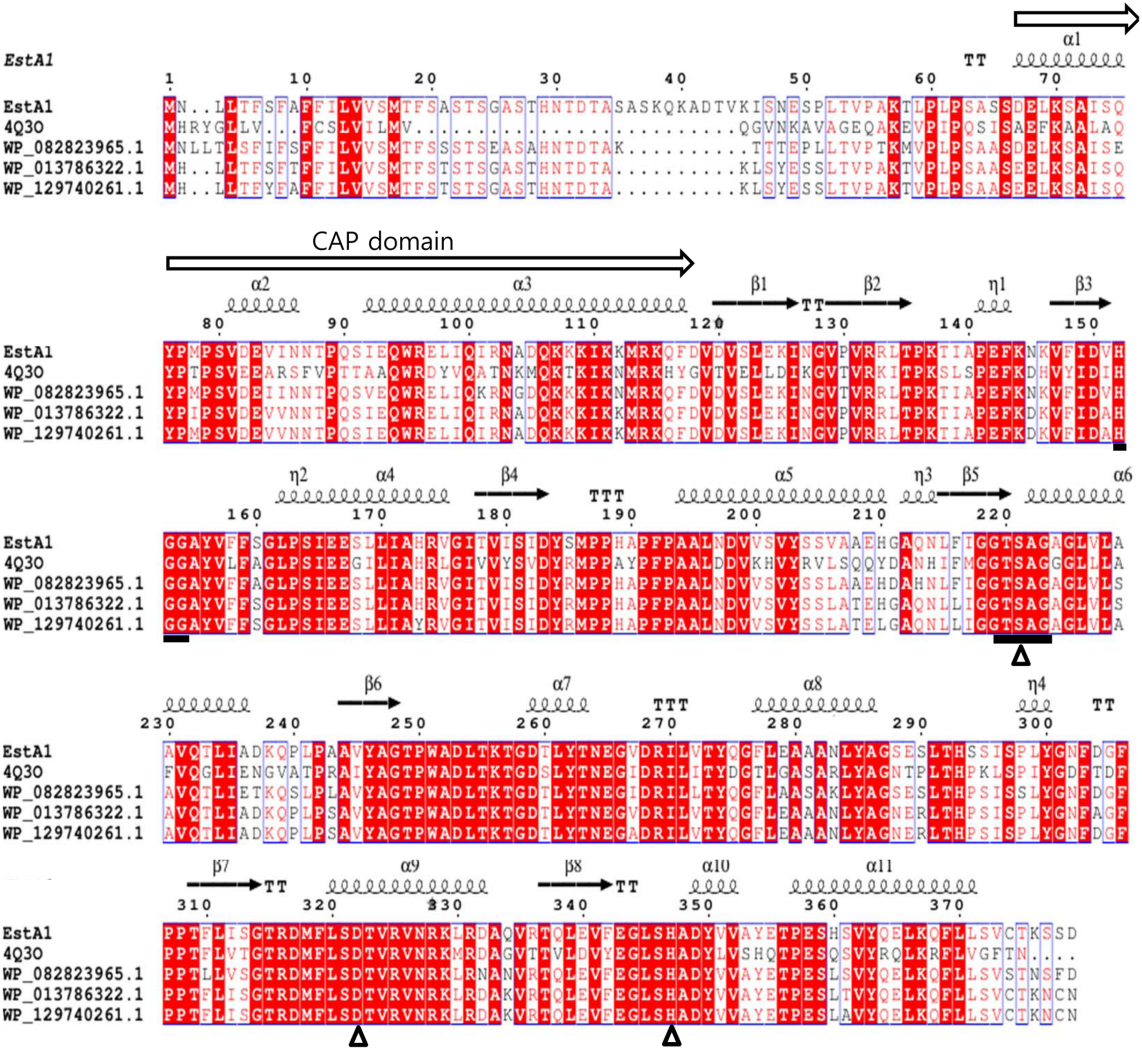
Multiple sequence alignment of esterase EstA1 with other related enzymes. 4Q3O, MGS-MT1 α/β hydrolase from uncultured bacteria; WP_082823965.1, α/β hydrolase from *Alteromonas stellipolaris*; WP_013786322.1, α/β hydrolase from *Alteromonas naphthalenivorans*; WP_129740261.1 α/β hydrolase from *Alteromonas* sp. 76-1. Empty triangles (△) represent putative catalytic triad: S221, D317, H347. The α-helix, β-sheet, random coil, beta turn represent as α, β, η, and T, respectively. Black squares represent the oxyanion hole and pentapeptide near active site serine residue. White arrow represents Cap domain of esterase EstA1. Red boxes indicate regions with identical amino acid residues.

As a result of multiple sequence alignment of the esterase EstAl with other esterases already identified in 3D structures, the conserved amino acid sequence (HGG and GTSAG motif) was confirmed and the catalytic triad was predicted as S221, D317 and H347 (Fig. 1).

To construct the structure homology model of esterase EstA1, we used the uncultured bacterium Mgs-mt1 esterase (PDB: 4Q3O) [30] as a template structure. As expected, the esterase EstA1 has an α/β hydrolase fold structure with a β sheet at the center and several α helixes surrounding back and forth (Fig. S5). The above-mentioned S221, D317 and H347 are located about 3Å away from each other at the active site pocket and are identified as catalytic triads (Fig. S5). Interestingly, family IV enzymes are composed of a cap domain covering the active site and a catalytic domain that occupies most of the rest. This cap domain was known to be directly or indirectly associated with enzyme activity, specificity, regioselectivity, thermophilicity and thermostability [8]. The structure of the esterase EstAl is clearly divided into cap domain consisting of three helices (α1, α2, and α3) and a catalytic domain containing the active site (Figs. 1 and S5).

### Expression and Purification of Recombinant Esterase EstA1

**Fig. 2:**
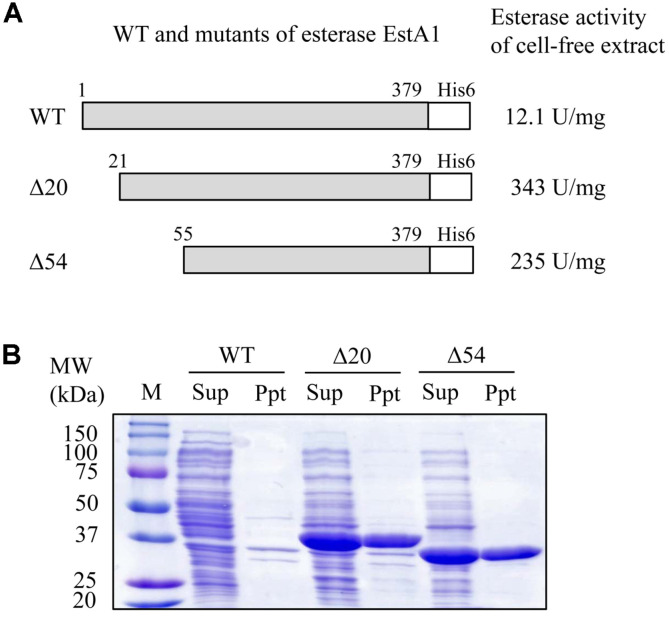
Construction of mutant esterase EstA1 for protein expression in *E. coli*. (**A**) Wild type (WT) and two mutants (Δ20, Δ54) were constructed and the esterase activities of the three recombinant *E. coli* cell-free extracts were measured. (**B**) Protein profiles of the three recombinant *E. coli* cells were analyzed by SDS-PAGE. M, protein size marker; Sup, supernatant (cell-free extract); Ppt, pellet.

The pET 22 vector was used to produce the esterase EstA1 in *E. coli* BL21 (DE3) cells. The intact protein with His6 tag at the C-terminal end was produced in *E. coli* cells, but the enzyme activity in cell-free extract was not high (12.1 U/mg protein) (Fig. 2A). Accordingly, the expected recombinant protein band was not detected in SDS-PAGE (Fig. 2B). To produce active esterase EstA1, we constructed two mutant proteins that partially removed its N-terminal sequences. The SignalP 4.1 Server program predicted that the N-terminal signal sequence was located from Met^1^ to Ser^20^, so we prepared one mutant enzyme with the signal sequence removed (Δ20). In addition, another mutant enzyme (Δ54) was prepared by removing N-terminal 54 amino acids by comparing with the Mgs-mt1 esterase (PDB: 4Q3O) which has already been revealed in its 3D structure. We tried to express the two mutants in *E. coli* BL21 (DE3) cells, and both showed significantly higher enzyme activity (343 U/mg for Δ20 mutant and 235 U/mg for Δ54 mutant) (Fig. 2A). We could also detect the expected sizes of the two soluble recombinant protein bands in SDS-PAGE (Fig. 2B). In this study, both mutants were purified and their biochemical characteristics were investigated.

**Fig. 3:**
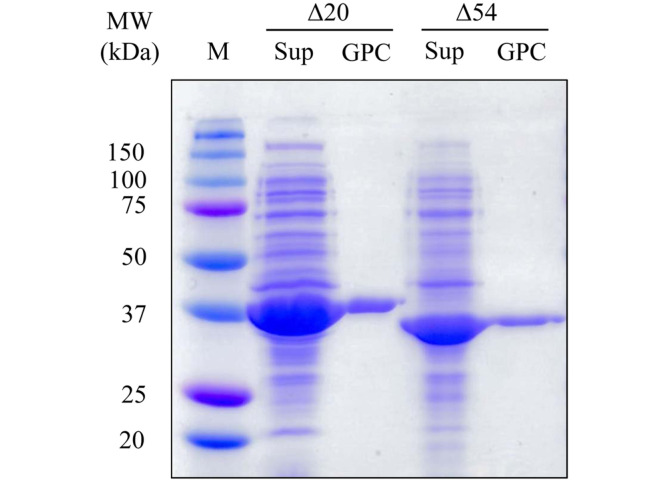
SDS-PAGE analysis of the esterase EstA1. Lane 1, protein size marker; Lane 2, cell-free extract of *E. coli* cells expressing Δ20 mutant; Lane 3, purified Δ20 mutant by Ni^2+^-NTA column and gel permeation chromatographies; Lane 4, cell-free extract of *E. coli* cells expressing Δ54 mutant; Lane 5, purified Δ54 mutant by Ni^2+^-NTA column and gel permeation chromatographies.

The mutant enzymes in *E. coli* cell-free extract were purified through Ni-NTA column chromatography and the residual imidazole was removed through gel permeation chromatography (Fig. 3). The specific activities of the finally purified mutant enzymes were 874 U/mg (Δ20 mutant) and 711 U/mg (Δ54 mutant) and the purification yields were calculated as 20% (Δ20 mutant) and 43% (Δ54 mutant)(Table S2).

### Characterization of the Δ20 and Δ54 Mutants

**Fig. 4:**
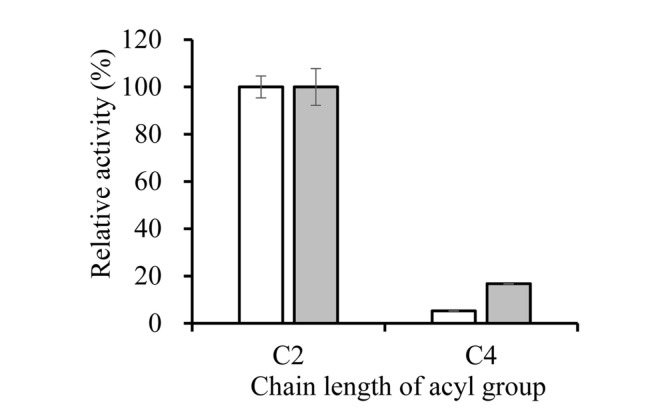
Substrate specificity of the Δ20 and Δ54 mutants. Hydrolytic activity of Δ20 and Δ54 mutant was measured using *p*NP acetate (C2) and *p*NP butyrate (C4). Open bar, Δ20 mutant; filled bar, Δ54 mutant.

The substrate specificity of the Δ20 and Δ54 mutants was determined using *p*NP-esters (C_2_-C_8_) as substrates in Tris-HCl buffer (pH 8.0) at 40°C (Fig. 4). Both Δ20 and Δ54 mutants hydrolyzed preferentially short-length acyl chain substrates. The highest specific activity was detected for *p*NP-acetate (C_2_) and enzyme activity for *p*NP-butyrate (C_4_) was less than 15% comparing with *p*NP-acetate. However, esterase EstA1 rarely hydrolyzed longer chain lengths (C_6_, C_8_) substrates. This feature indicates that both Δ20 and Δ54 mutants have very narrow substrate specificity.

**Fig. 5:**
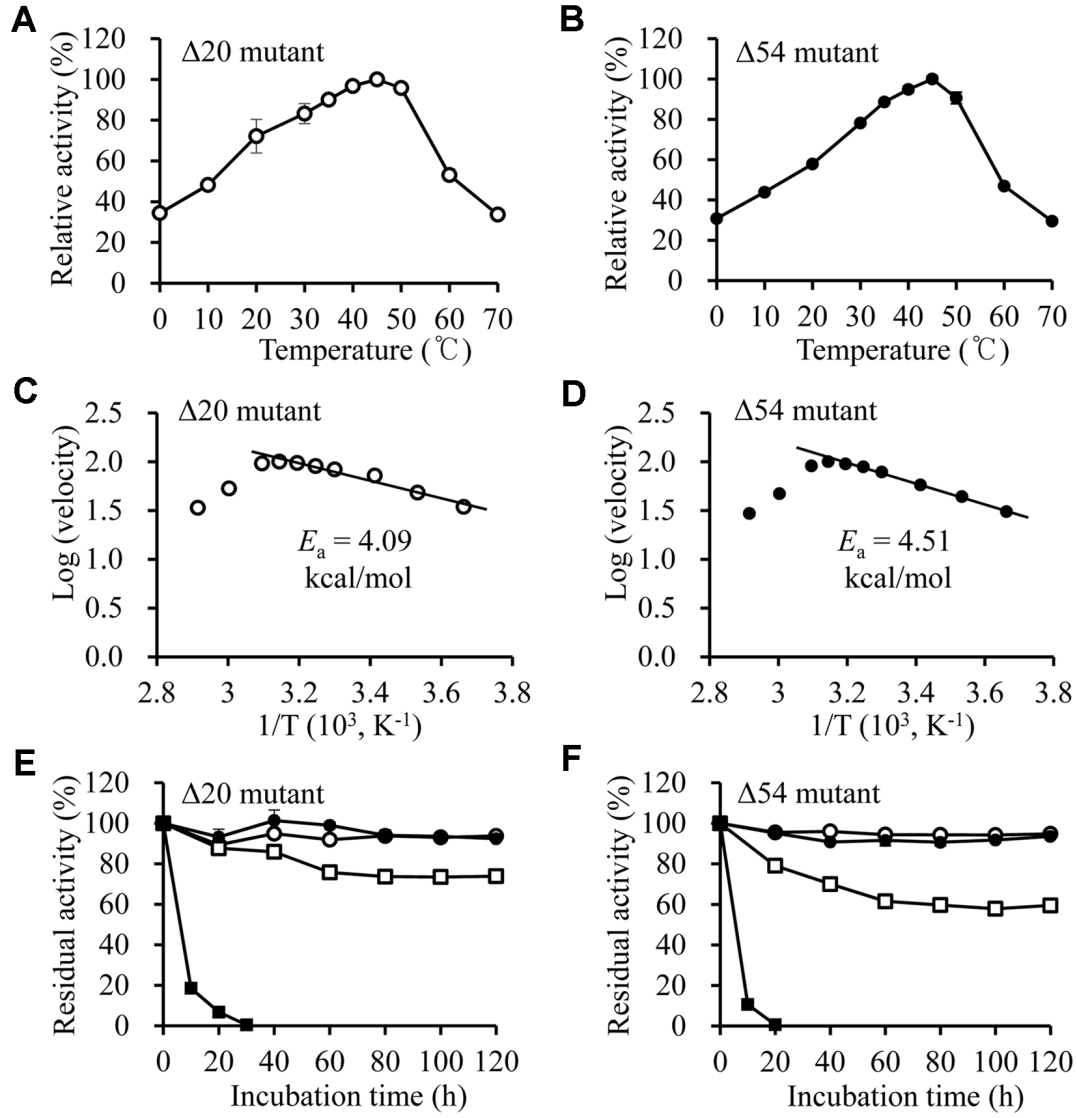
Effects of temperature on the Δ20 and Δ54 mutants. (**A** and **B**) The esterase activities of Δ20 and Δ54 mutants were measured at various temperatures. (**C** and **D**) Graphs were made using Arrhenius equation to obtain the activation energy of Δ20 and Δ54 mutant. (**E** and **F**) The residual activities of Δ20 and Δ54 mutants were measured after heattreating in the various temperatures. ○, 30°C; ●, 40°C; □, 50°C; ■, 55°C

Despite their preference for *p*NP-acetate, *p*NP-butyrate was used to analyze optimal temperature and pH, because *p*NP-acetate was unstable in the wide temperature and pH range. The effect of temperature on the Δ20 and Δ54 mutant activity was measured at 0-70°C (Figs. 5A and 5B). This enzyme was expected to be highly active at low temperatures because it was found in the Arctic Ocean, but the optimum reaction temperature was found to be 40°C when the relationship of temperature vs. activity was examined. Interestingly, they had 34% activity (Δ20) and 30% activity (Δ54) even at 0°C, which indicated that the enzymes could work at low temperatures such as in the Arctic region. In addition, the activation energies (E_a_) of the mutant enzymes were calculated as 4.09 kcal/mol (Δ20) and 4.51 kcal/mol (Δ54) in the temperature region from 0 to 40°C (Figs. 5C and 5D), which were very similar to the E_a_ values of typical cold-adapted enzymes [31]. Both Δ20 and Δ54 mutants were stable at 40°C for 120 h (Figs. 5E and 5F). At 50°C, the activity decreased to 74% (Δ20) and 59% (Δ54) after 120 h-incubation. At 55°C, most of the enzyme activity disappeared within 20 h, but the Δ20 mutant was found to be relatively thermo-stable compared to the Δ54 mutant.

**Fig. 6:**
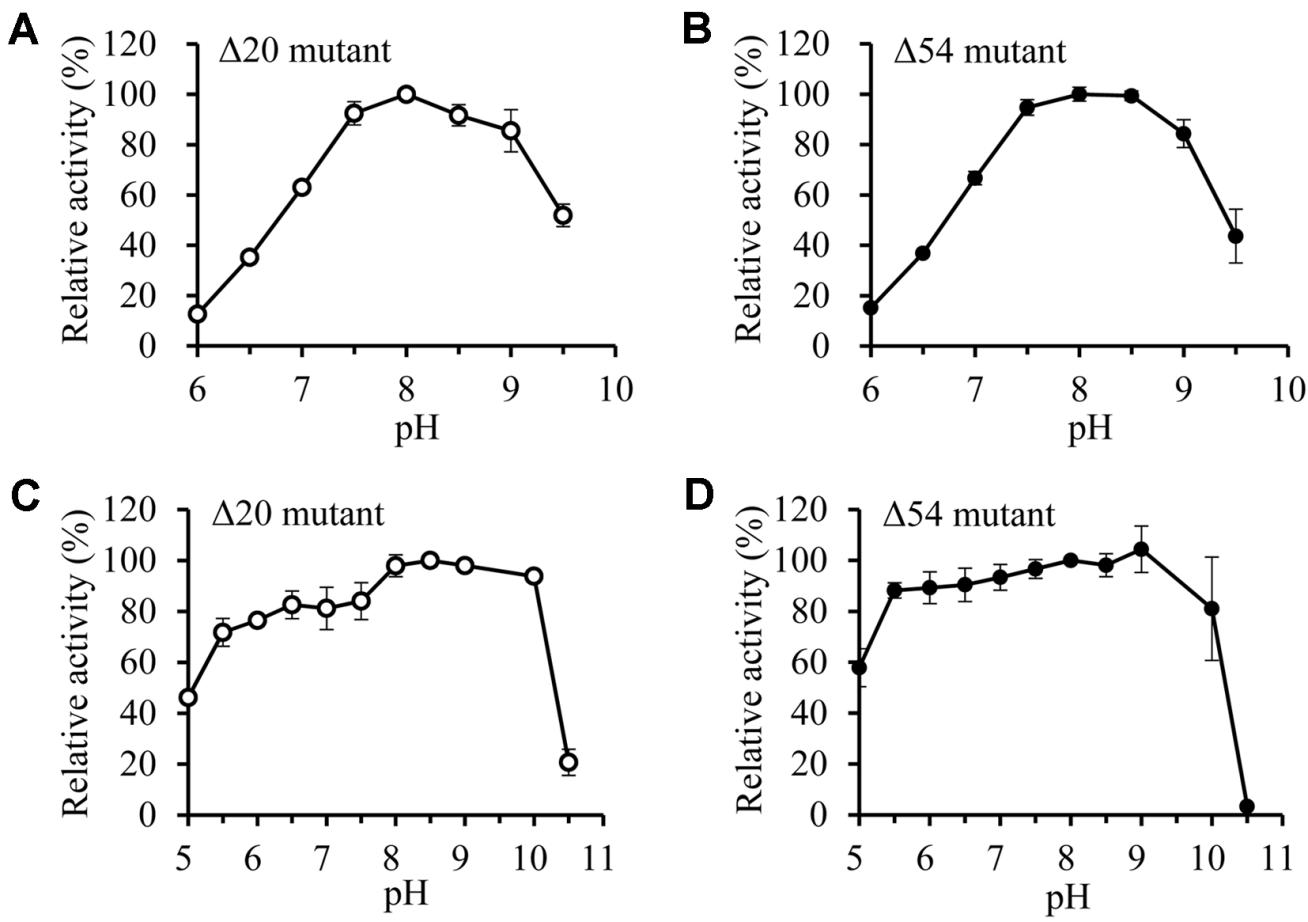
Effects of pH on the Δ20 and Δ54 mutants. (**A** and **B**) The esterase activities of Δ20 and Δ54 mutants were measured at various pHs. (**C** and **D**) The residual activities of Δ20 and Δ54 mutants were measured after 30 min-incubation in the various pH buffers. pH 5.0-6.0, 50 mM sodium acetate; pH 6.0-8.0, 50 mM potassium phosphate; pH 8.0-9.0, 50 mM Tris-HCl; pH 9.0-10.5, 50 mM carbonate-bicarbonate.

The effect of pH on the Δ20 and Δ54 mutant activity was measured at 40°C and pH 6.0-9.5 (Figs. 6A and 6B). Both mutant enzymes exhibited maximum activity at pH 8.0 and relatively high activity in the pH range of 7.5-9.0, suggesting that this enzyme is an alkaline esterase. For pH stability, both mutant enzymes were relatively stable in the pH range of pH 5.5-10.0. However, their enzyme stabilities decreased sharply when pH was higher than 10.0 (Figs. 6C and 6D).

### Effects of Organic Solvents, Metal Ions, Inhibitors, and Detergents on Δ20 and Δ54 Mutants

The activities of the Δ20 and Δ54 mutants were measured with several organic solvents, metal ions, inhibitors, and detergents. First, both mutants showed high stability up to 15% concentration of most organic solvents (Table 1). But they had reduced activity in acetonitrile solvent. When the concentration of organic solvents increased to 30%, this enzyme showed stability only in DMSO, methanol, cyclohexane, and n-hexane. When the concentration of organic solvents increased to 50%, the mutant enzymes still showed high stability in DMSO, cyclohexane, and n-hexane, suggesting that these enzymes are organic solvent-tolerant enzymes.

Both mutant enzymes exhibited high activity on many metal ions. The enzyme activity was maintained at the concentrations of 1 mM and 5 mM of metal ions (Li^+^, Na^+^, K^+^, Mg^2+^, Ca^2+^, Co^2+^, and Ni^+^), however, enzyme activity was significantly reduced at the concentrations of 5 mM of some metal ions (Zn^2+^ and Cu^2+^) (Table 2). These ions are expected to have the ability to inhibit the enzyme activity of esterase/lipase. Enzyme activity of metagenome esterase E25 [32], metagenome esterase E40 [33], *Alkalibacterium* esterase rEstSL3 [34], and metagenome esterase H8 [35] tended to decrease in the presence of these metal ions, indicating that they are potential inhibitors to esterase/lipase.

**Fig. 7:**
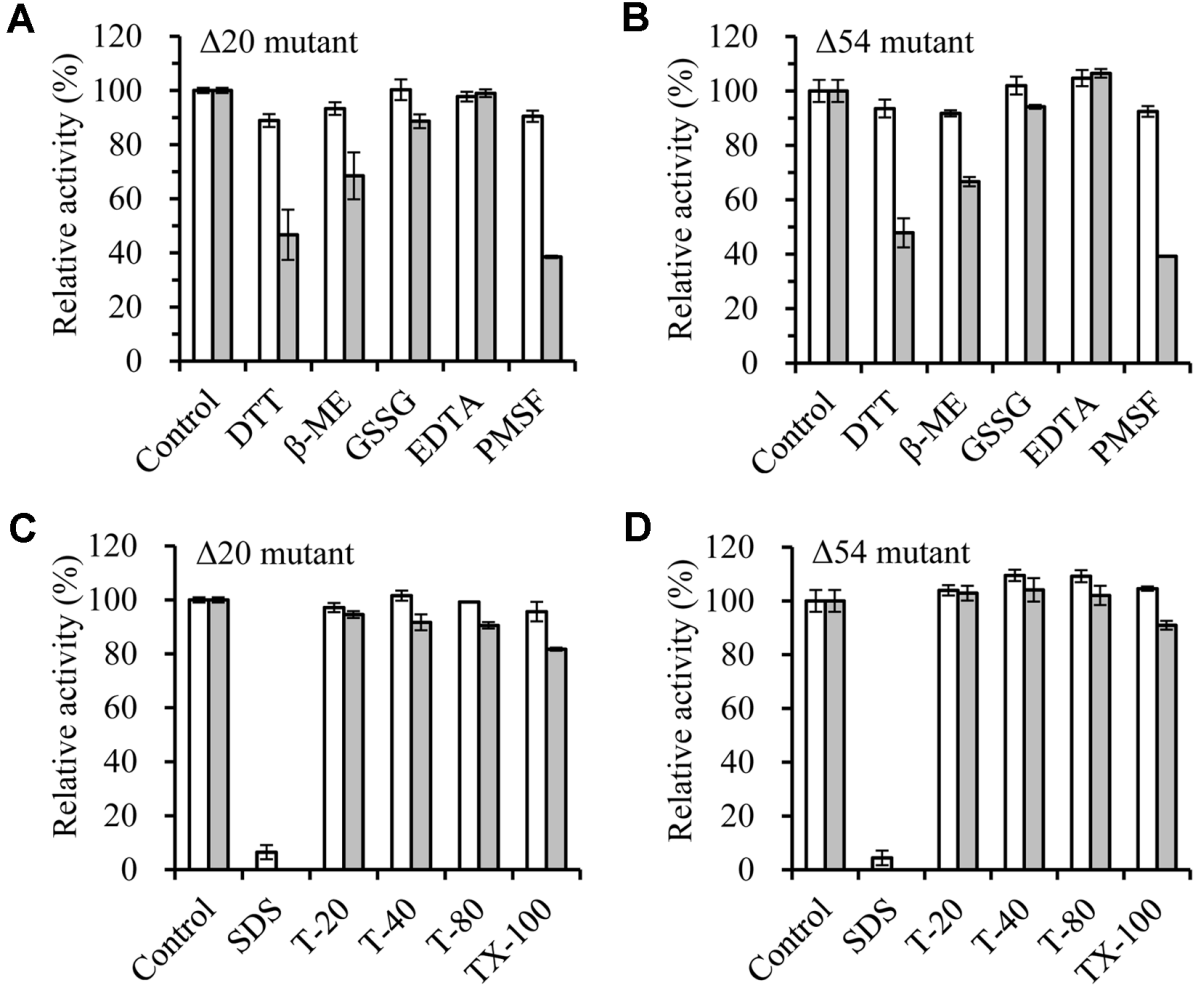
Effects of inhibitor and detergent on the Δ20 and Δ54 mutants. (**A** and **B**) The residual activities of Δ20 and Δ54 mutants were measured after incubation in various inhibitors. Open bar, 1 mM; closed bar, 5 mM. (**C** and **D**) The residual activities of Δ20 and Δ54 mutants were measured after incubation in various detergents. Open bar, 0.1%; filled bar, 0.5%.

Their enzyme activities were maintained against some chemical agents (DTT, β-ME, GSSG, EDTA, and PMSF) at low concentration (1 mM) (Figs. 7A and 7B). However, their activities were significantly reduced against reducing agents (DTT and β-ME) and serine protease inhibitor (PMSF) at high concentration (5 mM), indicating that serine residue is present in the active site of this enzyme. These enzymes exhibited high stability at concentration of 0.1%and 0.5% of most neutral detergents including T-20, T-40, T-80, and TX-100. But the enzyme activity was greatly reduced by 0.1% concentration of SDS, an anionic detergent (Figs. 7C and 7D).

### Effect of NaCl on Δ20 and Δ54 Mutant Activity and Stability

**Fig. 8:**
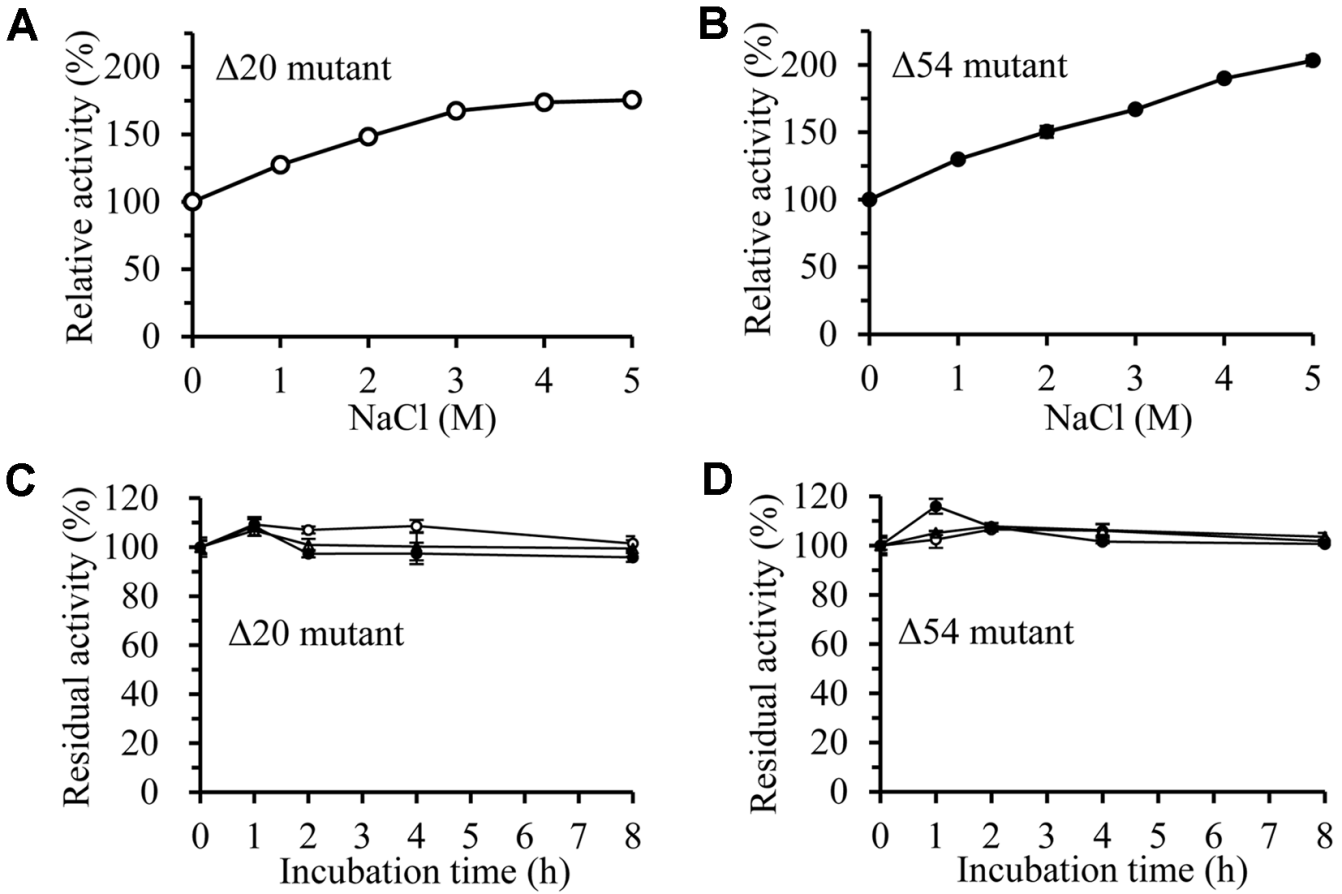
Effects of NaCl on the Δ20 and Δ54 mutants. (**A** and **B**) The esterase activities of Δ20 and Δ54 mutants were measured at various NaCl concentrations. (**C** and **D**) The residual activities of Δ20 and Δ54 mutants were measured after incubation in NaCl solution. ○, 3 M NaCl; ●, 4 M NaCl; △, 5 M NaCl.

Since the enzyme was isolated from Arctic marine microorganisms, it was expected to have stability and activity against high concentration of NaCl. The activities of the Δ20 and Δ54 mutants were continuously increased with increasing NaCl concentration and enzyme activities were measured to be 175% (Δ20) and 203% (Δ54) at 5 M NaCl concentration (Figs. 8A and 8B). This enzyme maintained its activity for 8 h in the presence of 3 M, 4 M, and 5 M NaCl (Figs. 8C and 8D). Water molecules are essential for protein conformation and function and prevent protein aggregation [36]. High concentrations of salt ions in aqueous solutions reduce the number of water molecules available to the protein, making it impossible for the protein to be hydrated [37]. This phenomenon is analogous to the reduction of water molecules available to proteins by organic solvents. However, halophilic proteins can retain their structure and maintain their enzymatic activity even when the salt ions are increased [23]. Some halophilic enzymes may exhibit better activity under high salt concentration. Salt concentrations may significantly affect the folding, conformation, subunit structure, and kinetics of halophilic proteins. Since this enzyme has high activity and stability in high salt concentration, the study of its structure and activity is considered to be academically and industrially meaningful.

Taken together, new enzyme esterase EstA1 was isolated from the Arctic *Alteromonas* sp. 39-G1 strain. We failed to express the wild type of this enzyme in *E. coli*, but succeeded in producing mutant enzymes (Δ20 and Δ54) as active forms. Both Δ20 and Δ54 mutants were purified and characterized for the first time in this research. These mutant enzymes were found to have cold-adapted, alkaline, and salt-tolerant properties, so we think that they are very likely to be used academically and industrially.

## Supplemental Materials



Supplementary data for this paper are available on-line only at http://jmb.or.kr.

## Figures and Tables

**Table 1:** Effects of organic solvents on the EstA1 esterase.

Solvent	logP	Δ20 mutant			Δ54 mutant		

		15%	30%	50%	15%	30%	50%
None		100	100	100	100	100	100
DMSO	-1.3	106	101	98	81	101	88
Methanol	-0.76	100	94	ND	83	71	ND
Acetonitrile	-0.33	68	ND	ND	39	ND	ND
Ethanol	-0.24	87	7	ND	86	ND	ND
Acetone	-0.23	108	66	ND	112	55	ND
Isopropanol	0.1	102	ND	ND	103	ND	ND
Cyclohexane	3.2	101	131	135	119	127	147
n-Hexane	3.5	103	121	159	108	119	131

^*^ND, not detected

**Table 2:** Effects of metal ions on the EstA1 esterase.

Ion	Δ20 mutant		Δ54 mutant	

	1mM	5mM	1mM	5mM
None	100	100	100	100
LiCl	102	100	101	101
NaCl	96	93	103	103
K_2_SO_4_	101	103	101	104
MgCl_2_	98	101	102	101
CaCl_2_	101	99	104	103
CoCl_2_	99	101	96	109
NiSO_4_	99	97	97	89
ZnSO_4_	92	79	87	58
CuSO_4_	89	79	90	69
