# Supplementary material for: Characterization of Novel Salt-Tolerant Esterase Isolated from the Marine Bacterium Alteromonas sp. 39-G1
Source: J Microbiol Biotechnol. 2019 Dec 2;30(2):216–25. doi: 10.4014/jmb.1907.07057 (PMC9728341; doi:10.4014/jmb.1907.07057)
Supplement: Supplementary file 1 [file JMB-30-2-216-supple.pdf]

Journal of Microbiology and Biotechnology

J. Microbiol. Biotechnol.
https://doi.org/10.4014/jmb.1907.07057

Supplementary Table S1. Primer sequences used in this experiments

Cloning gene	Primer sequence
16s rRNA	27F 5'-AGA GTT TGA TCM TGG CTC AG-3'
	1492R 5'-GGT TAC CTT GTT ACG ACT T-3'
WT EstA1	Forward primer: 5'-CATATGAACCTACTTACCTTTTCC-3'
	Reverse primer: 5'-CTCGAGGTCGCTACTTTTAGTACACA-3'
mutant (Δ20)	Forward primer: 5'-CATATGTCTACATCTGGGGCTTCT-3'
	Reverse primer: 5'-CTCGAGGTCGCTACTTTTAGTACACA-3'
mutant (Δ54)	Forward primer: 5'-CATATGGCTAAAACGTTACCGCTG-3'
	Reverse primer: 5'-CTCGAGGTCGCTACTTTTAGTACACA-3'

Supplementary Table S2. Purification of EstA1

	Purification step	Total activity (U)	Total protein (mg)	Specific activity (U/mg)	Purification fold	Recovery (%)
Δ20	Cell-free extract	40100	117	343	1.00	100
	Ni-NTA agarose	14300	23.9	598	1.7 4	35.7
	Superose 12	8180	9.36	874	2.55	20.4
Δ54	Cell-free extract	12100	51.5	235	1.00	100
	Ni-NTA agarose	9640	16.5	584	2.49	79.7
	Superose 12	5220	7.34	711	3.0 3	43.1

```
1    ATTGAACGCTGGCGGCAGGCCTAACACATGCAAGTCGAACGGAAACATGTCTAGCTTGCT    60
61   AGATGATGTCGAGTGGCGGACGGGTGAGTAATGCTTGGGAACTTGCCTTTGCGAGGGGGA   120
121  TAACAGTTGGAAACGACTGCTAATACCGCATAATGTCTTCGGACCAAACGGGGCTTAGGC   180
181  TCCGGCGCAAAGAGAGGCCCAAGTGAGATTAGCTAGTTGGTGAGGTAAAGGCTCACCAAG   240
241  GCAACGATCTCTAGCTGTTCTGAGAGGAAGATCAGCCACACTGGGACTGAGACACGGCCC   300
301  AGACTCCTACGGGAGGCAGCAGTGGGGAATATTGCACAATGGGGGAAACCCTGATGCAGC   360
361  CATGCCGCGTGTGTGAAGAAGGCCTTCGGGTTGTAAAGCACTTTCAGTTGTGAGGAAAAG   420
421  TTAGTAGTTAATACCTGCTAGCCGTGACGTTAACAACAGAAGAAGCACCGGCTAACTCCG   480
481  TGCCAGCAGCCGCGGTAATACGGAGGGTGCGAGCGTTAATCGGAATTACTGGGCGTAAGC   540
541  GCACGCAGGCGGTTTGTTAAGCTAGATGTGAAAGCCCCGGGCTCAACCTGGGACGGTCAT   600
601  TTAGAACTGGCAGACTAGAGTCTTGGAGAGGGGAGTGGAATTCCAGGTGTAGCGGTGAAA   660
661  TGCGTAGATATCTGGAGGAACATCAGTGGCGAAGGCGACTCCCTGGCCAAAGACTGACGA   720
721  CTCATGTGCGAAAGTGTGGGTAGCGAACAGGATTAGATACCCTGGTAGTCCACACCGTAA   780
781  ACGCTGTCTACTAGCTGTTTGTGGCTTTAAGCCGTGAGTAGCGAAGCTAACGCGATAAGT   840
841  AGACCGCCTGGGGAGTACGGCCGCAAGGTTAAAACTCAAATGAATTGACGGGGGCCCGCA   900
901  CAAGCGGTGGAGCATGTGGTTTAATTCGATGCAACGCGAAGAACCTTACCTACACTTGAC  1060
1061 ATGTTGAGAAGTTACTAGAGATAGTTTCGTGCCTTCGGGAACTCAAACACAGGTGCTGCA  1120
1121 TGGCTGTCGTCAGCTCGTGTCGTGAGATGTTGGGTTAAGTCCCGCAACGAGCGCAACCCT  1180
1181 TGTCCTTAGTTGCCAGCATTTAGTTGGGCACTCTAAGGAGACTGCCGGTGACAAACCGGA  1240
1241 GGAAGGTGGGGACGACGTCAAGTCATCATGGCCCTTACGTGTAGGGCTACACACGTGCTA  1300
1301 CAATGGCATATACAGAGGGATGCGAGACAGTGATGTGGAGCGGACCCCTTAAAGTATGTC  1360
1361 GTAGTCCGGATTGGAGTCTGCAACTCGACTCCATGAAGTCGGAATCGCTAGTAATCGCAG  1420
1421 GTCAGAATACTGCGGTGAATACGTTCCCGGGCCTTGTACACACCGCCCGTCACACCATGG  1480
1481 GAGTGGGATGCAAAAGAAGTAGTTA                                     1505
```

Supplementary Fig. S1. 16S rRNA sequence of strain 39-G1

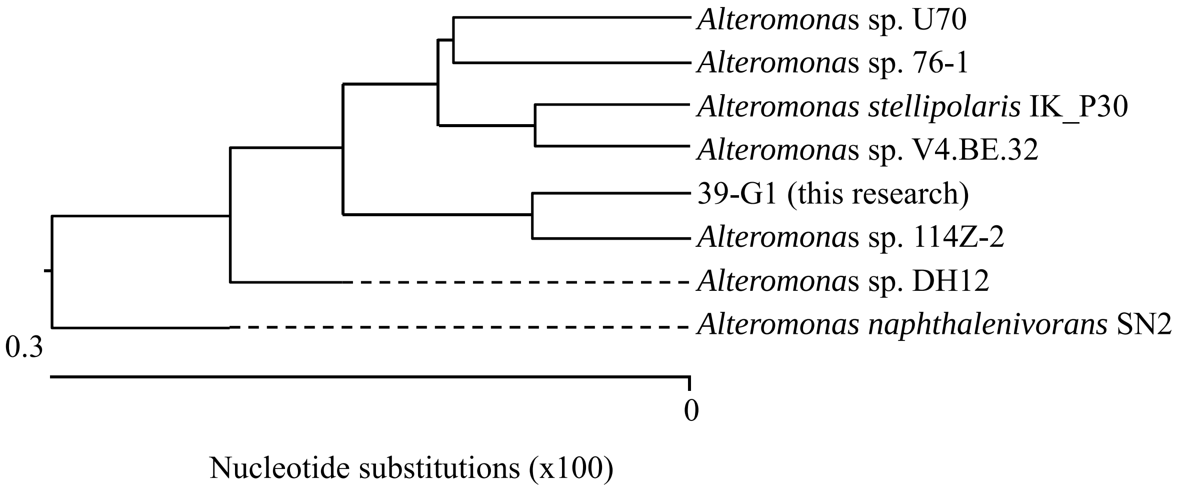

Supplementary Fig. S2. Phylogenetic tree of strain 39-G1 using 16S rRNA sequence. The 16S rRNA sequence of this strain had 99.86% homology with that of *Alteromonas* sp. 76-1 (GenBank: KY697829.1), 99.86% with *Alteromonas naphthalenivorans* SN2 (GenBank: GU166736.2), and 99.64% with *Alteromonas stellipolaris* IK_P30 (GenBank: LR218081.1).

```
   1  ATGAACCTACTTACCTTTTCCTTCGCCTTTTTCATTTTGGTGGTATCAATGACCTTTTCGGCTTCTACATCTGGGGCTTCTACCCACAAC    90
   1   M  N  L  L  T  F  S  F  A  F  F  I  L  V  V  S  M  T  F  S  A  S  T  S  G  A  S  T  H  N    30
  91  ACTGATACGGCCAGTGCGAGTAAACAAAAGGCCGATACAGTCAAAATCTCTAATGAATCTCCGTTAACCGTGCCAGCTAAAACGTTACCG   180
  31   T  D  T  A  S  A  S  K  Q  K  A  D  T  V  K  I  S  N  E  S  P  L  T  V  P  A  K  T  L  P    60
 181  CTGCCAAGTGCTTCAAGCGATGAACTTAAAAGCGCGATTTCTCAATACCCCATGCCGTCAGTTGATGAAGTCATTAACAACACGCCTCAA   270
  61   L  P  S  A  S  S  D  E  L  K  S  A  I  S  Q  Y  P  M  P  S  V  D  E  V  I  N  N  T  P  Q    90
 271  TCTATAGAGCAGTGGCGTGAATTGATTCAAATAAGAAATGCAGATCAGAAAAAGAAAATCAAAAAAATGCGCAAGCAGTTTGATGTTGAT   360
  91   S  I  E  Q  W  R  E  L  I  Q  I  R  N  A  D  Q  K  K  K  I  K  K  M  R  K  Q  F  D  V  D   120
 361  GTGTCTTTGGAAAAAATTAATGGCGTACCGGTCAGGCGACTTACGCCAAAGACCATTGCCCCTGAATTTAAAAACAAGGTATTTATTGAC   450
 121   V  S  L  E  K  I  N  G  V  P  V  R  R  L  T  P  K  T  I  A  P  E  F  K  N  K  V  F  I  D   150
 451  GTACACGGTGGCGCTTACGTGTTTTTTTCCGGGCTGCCCAGCATAGAAGAAAGCTTGTTAATTGCCCATAGAGTGGGTATTACAGTGATT   540
 151   V  H  G  G  A  Y  V  F  F  S  G  L  P  S  I  E  E  S  L  L  I  A  H  R  V  G  I  T  V  I   180
 541  AGTATCGATTATAGCATGCCACCTCATGCGCCATTTCCAGCAGCACTTAACGATGTAGTGTCGGTTTATAGCAGCGTGGCTGCAGAACAT   630
 181   S  I  D  Y  S  M  P  P  H  A  P  F  P  A  A  L  N  D  V  V  S  V  Y  S  S  V  A  A  E  H   210
 631  GGCGCACAGAATCTTTTTATAGGTGGCACATCTGCAGGAGCAGGTTTAGTGCTTGCTGCCGTGCAAACGCTTATTGCTGACAAGCAACCG   720
 211   G  A  Q  N  L  F  I  G  G  T  S  A  G  A  G  L  V  L  A  A  V  Q  T  L  I  A  D  K  Q  P   240
 721  TTGCCAGCAGCAGTGTATGCGGGCACCCCTTGGGCTGATTTGACGAAAACCGGCGATACCCTTTATACCAATGAAGGTGTTGATCGTATT   810
 241   L  P  A  A  V  Y  A  G  T  P  W  A  D  L  T  K  T  G  D  T  L  Y  T  N  E  G  V  D  R  I   270
 811  TTGGTGACATACCAAGGGTTTTTGGAAGCAGCGGCTAATCTATATGCTGGAAGCGAAAGCTTAACCCACTCCTCTATTTCCCCTCTTTAT   900
 271   L  V  T  Y  Q  G  F  L  E  A  A  A  N  L  Y  A  G  S  E  S  L  T  H  S  S  I  S  P  L  Y   300
 901  GGCAATTTTGATGGCTTCCCGCCTACGTTTTTAATATCTGGCACGCGAGATATGTTTCTAAGCGATACGGTGCGGGTGAATCGTAAGCTA   990
 301   G  N  F  D  G  F  P  P  T  F  L  I  S  G  T  R  D  M  F  L  S  D  T  V  R  V  N  R  K  L   330
 991  CGTGATGCACAAGTACGCACCCAGTTAGAGGTTTTCGAAGGGTTATCCCATGCCGATTACGTTGTTGCTTATGAAACCCCAGAATCTCAC  1080
 331   R  D  A  Q  V  R  T  Q  L  E  V  F  E  G  L  S  H  A  D  Y  V  V  A  Y  E  T  P  E  S  H   360
1081  TCTGTATACCAAGAGCTTAAGCAATTTCTACTAAGCGTGTGTACTAAAAGTAGCGACTAA                                 1140
 361   S  V  Y  Q  E  L  K  Q  F  L  L  S  V  C  T  K  S  S  D ***                                  379
```

Supplementary Fig. S3. Nucleotide and protein sequence of esterase EstA1

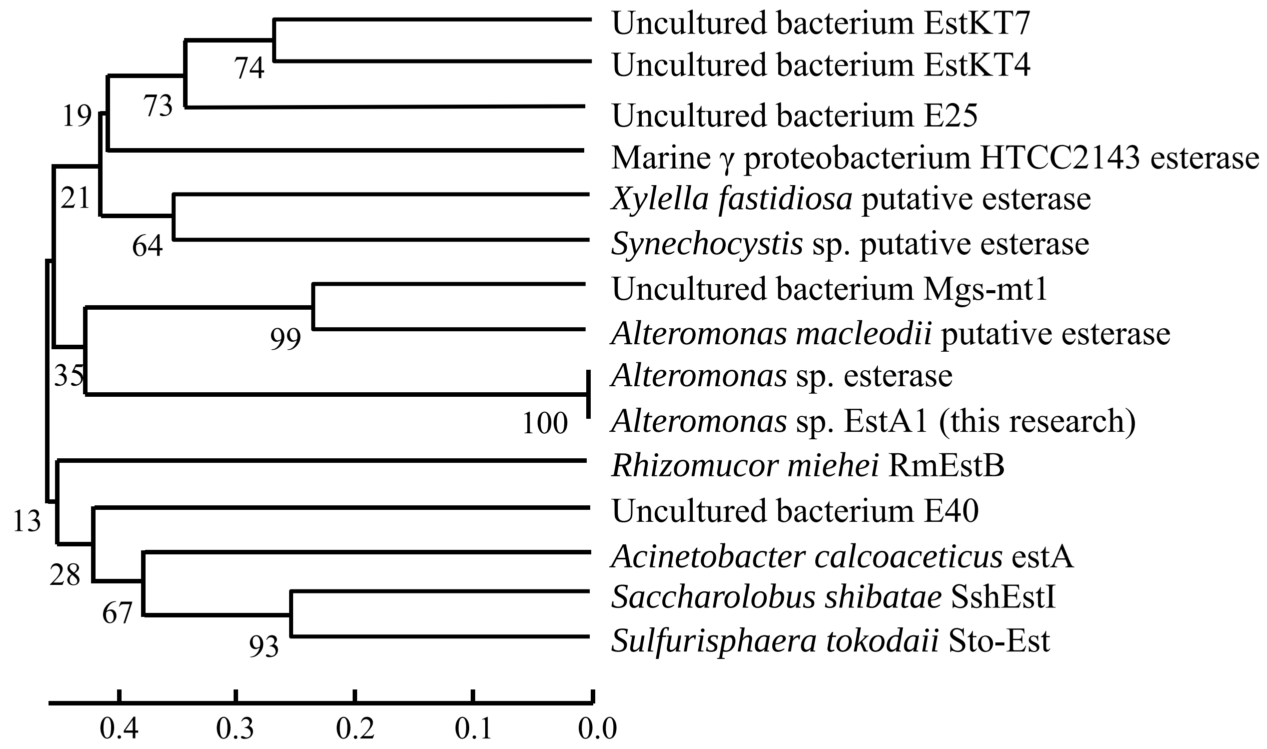

Supplementary Fig. S4. Phylogenetic analysis of esterase EstA1 with closely related family IV proteins. Esterase EstA1 sequence was compared with similar proteins including uncultured bacterium EstKT7 (GenBank ADH59413.1), uncultured bacterium EstKT4 (GenBank ADH59412.1), uncultured bacterium E25 (PDB 4Q05), marine gamma proteobacterium HTCC2143 putative esterase (ZP_01615954.1), *Xylella fastidiosa* putative esterase (UniProtKB/Swiss-Prot Q9PCN5), *Synechocystis* sp. putative esterase (UniProtKB/Swiss-Prot P72953), uncultured bacterium Mgs-mt1 (PDB 4Q3O), *Alteromonas macleodii* esterase (YP_002127854.1), *Alteromonas* sp. esterase (GenBank PHS53692.1), *Rhizomucor miehei* RmEstB (PDB 4WY8), uncultured bacterium E40 (PDB 4XVC), *Acinetobacter calcoaceticus* estA (GenBank CAA50601.1), *Saccharolobus shibatae* SshEstI (PDB 3WJ1), *Sulfurisphaera tokodaii* Sto-Est (PDB 3AIK). The phylogenetic tree was generated with the neighbor-joining method (MEGA 5.2). Bootstrap values (n = 1,000 replicates) are reported as percentages.

A B C

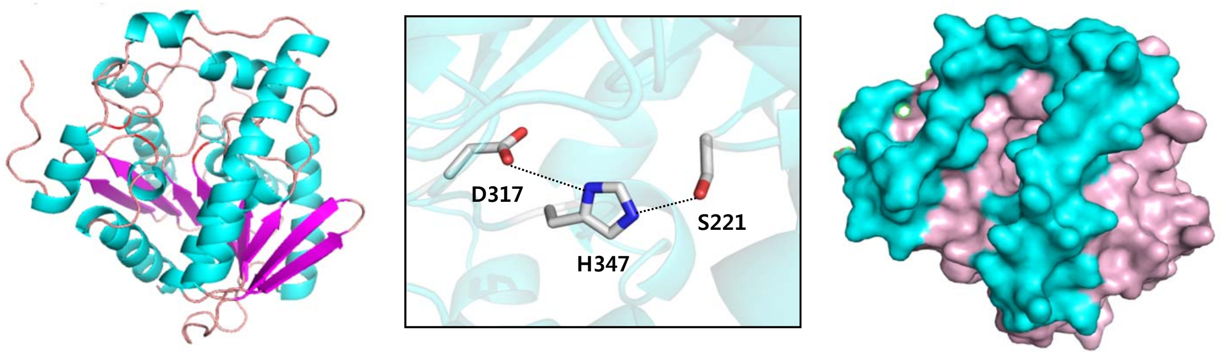

Supplementary Fig. S5. Structure homology model of esterase EstA1 A, Homology model of esterase EstA1 constructed by SWISS-MODEL with MGS-MT1 (PDB 4Q3O) used as the template. The α-helix, β-sheet, loop and catalytic triad are shown cartoon in sky blue, purple, pink, red, respectively. B, An enlarged view of the esterase EstA1 putative catalytic triad. Three amino acids (S221, D317, H347) that were expected to affect enzyme activity were located close to each other. C, Cap domain and catalytic domain are shown purple and red, respectively.
